# Ketamine administration in idiopathic epileptic and healthy control dogs: Can we detect differences in brain metabolite response with spectroscopy?

**DOI:** 10.3389/fvets.2022.1093267

**Published:** 2023-01-06

**Authors:** Manuela Wieser, Katrin Melanie Beckmann, Annette P. N. Kutter, Nico Mauri, Henning Richter, Niklaus Zölch, Rima Nadine Bektas

**Affiliations:** ^1^Section of Anesthesiology, Department of Clinical Diagnostics and Services, University of Zurich, Zurich, Switzerland; ^2^Section of Neurology, Clinic of Small Animals, University of Zurich, Zurich, Switzerland; ^3^Department of Clinical Diagnostics and Services, Clinic for Diagnostic Imaging, University of Zurich, Zurich, Switzerland; ^4^Vetimage Diagnostik AG, Oberentfelden, Switzerland; ^5^Department of Forensic Medicine and Imaging, Institute of Forensic Medicine, University of Zurich, Zurich, Switzerland

**Keywords:** spectroscopy, glutamate, GABA, glucose, MRS, GLX, thalamus, seizures

## Abstract

**Introduction:**

In recent years ketamine has increasingly become the focus of multimodal emergency management for epileptic seizures. However, little is known about the effect of ketamine on brain metabolites in epileptic patients. Magnetic resonance spectroscopy (MRS) is a non-invasive technique to estimate brain metabolites *in vivo*. Our aim was to measure the effect of ketamine on thalamic metabolites in idiopathic epileptic (IE) dogs using 3 Tesla MRS. We hypothesized that ketamine would increase the glutamine—glutamate (GLX)/creatine ratio in epileptic dogs with and without antiseizure drug treatment, but not in control dogs. Furthermore, we hypothesized that no different responses after ketamine administration in other measured brain metabolite ratios between the different groups would be detected.

**Methods:**

In this controlled prospective experimental trial IE dogs with or without antiseizure drug treatment and healthy client-owned relatives of the breeds Border Collie and Greater Swiss Mountain Dog, were included. After sedation with butorphanol, induction with propofol and maintenance with sevoflurane in oxygen and air, a single voxel MRS at the level of the thalamus was performed before and 2 min after intravenous administration of 1 mg/kg ketamine. An automated data processing spectral fitting linear combination model algorithm was used to estimate all commonly measured metabolite ratios. A mixed ANOVA with the independent variables ketamine administration and group allocation was performed for all measured metabolites. A *p* < 0.05 was considered statistically significant.

**Results:**

Twelve healthy control dogs, 10 untreated IE and 12 treated IE dogs were included. No significant effects for GLX/creatine were found. However, increased glucose/creatine ratios were found (*p* < 0.001) with no effect of group allocation. Furthermore, increases in the GABA/creatine ratio were found in IEU dogs.

**Discussion:**

MRS was able to detect changes in metabolite/creatine ratios after intravenous administration of 1 mg/kg ketamine in dogs and no evidence was found that excitatory effects are induced in the thalamus. Although it is beyond the scope of this study to investigate the antiseizure potential of ketamine in dogs, results of this research suggest that the effect of ketamine on the brain metabolites could be dependent on the concentrations of brain metabolites before administration.

## 1. Introduction

The use of ketamine in canine epilepsy has undergone a paradigm shift in recent years. Historically, it has been suggested to avoid ketamine in epileptic humans and dogs due to its proconvulsive properties (1–5), but in recent years, ketamine has been increasingly promoted as a game changer for status epilepticus in human (6–9) as well as in veterinary medicine (10–12).

Ketamine exerts its action over non-competitive N-methyl-d-aspartate (NMDA) receptor antagonism (13–15). In the brain, these receptors are located in the cerebral cortex, thalamus and hippocampus (16–19). NMDA receptors are glutamate receptors and are expressed as autoreceptors or as heteroreceptors. In autoreceptors there is a binding with a specific ligand, whereas in heteroreceptors neurotransmitters from other neurons can bind. NMDA heteroreceptors might modulate not only glutamate release but also the release of γ-aminobutyric acid (GABA) (20), noradrenaline (21, 22) or dopamine (23). During status epilepticus an up-regulation of NMDA receptors in the brain takes place (24). Blockage of these NMDA receptors through ketamine can inhibit conduction of excitation and could therefore resolve status epilepticus (25).

The exact etiology of the proconvulsant effect of ketamine remains unclear (26). The avoidance of ketamine in dogs suffering from epilepsy has been based on limited evidence (2, 3). It has been postulated by Ferrer-Allado et al. (2) that doses of ketamine between 2 and 4 mg/kg intravenous (IV) caused electrical seizure activity. This electroconvulsive phenomenon coincided with the peak concentration of ketamine in the arterial blood. This finding, together with case reports of dogs experiencing seizure after ketamine administration, most likely contributed to the fact that people were reluctant to use ketamine in epileptic animals (4, 5).

Not only during the epileptic seizure activity but also in between epileptic seizures, in the interictal period, the epileptic brain shows functional and metabolic abnormalities (27–30) and these changes might differ between treated and non-treated epileptic dogs (31). Evidence for such a difference comes from recent studies. In idiopathic epileptic (IE) dogs significantly lower GABA levels were found in urine specimens in untreated compared to treated dogs (32). Besides these indirect metabolite measurements in the urine, lower glutamine—glutamate (GLX)/creatine ratios have been detected in the brain of IE dogs under antiseizure drug treatment compared to those without antiseizure drug treatment and to healthy control dogs (31, 32).

In human medicine ketamine has been shown to have different effects on brain metabolites in healthy people than in those suffering from functional brain diseases such as depression or compulsive behavioral disorders (33, 34). Those different effects were detected by using proton magnetic resonance spectroscopy (^1^H-MRS). It is suspected that not only a pre-existing abnormal neurotransmitter level of GABA or glutamate can cause different changes in the response of brain metabolites after ketamine administration, but that also different doses of ketamine can affect neurotransmitter levels (35).

Single voxel ^1^H-MRS enables non-invasive *in vivo* measurement of metabolites ratios or concentrations in a selected volume of interest within the brain and allows detection of changes in metabolites after drug administration (36). ^1^H-MRS allows, among others, the measurement of glutamate, glutamine and GABA (37). At 3 Tesla (T) glutamate and glutamine are commonly reported together as GLX due to the overlap of glutamate and glutamine spectra and the difficulties in separating these two spectra at this field strength (38). Furthermore, also GABA measurements by 3 T MRS have to be interpreted with caution, due to the low concentration of GABA in the brain and the spectral overlap with other metabolites (39, 40).

In humans and experimental animals MRS has been used to investigate the effect of multiple drugs in health and disease (41–43). In the canine population, MRS has been used to evaluate metabolic changes in IE dogs (31, 44) and to investigate the effect of anesthetic drugs on brain metabolites in healthy epilepsy unaffected animals (45, 46). However, studies investigating the effect of drugs in IE are currently lacking.

The aim of this study was to compare metabolites in the thalamus before and after IV administration of 1 mg/kg ketamine in IE dogs with and without antiseizure drug treatment and in their not affected relatives using single voxel MRS.

We hypothesized that a bolus of ketamine would increase GLX/creatine ratio in the thalamus in epileptic dogs with and without antiseizure drug treatment, but not in control dogs. Furthermore, we hypothesized that the change of the other commonly measured metabolite ratios would not be different between groups after 1 mg/kg ketamine administration.

## 2. Materials and methods

The study was performed as a prospective experimental trial. This project was approved by the local ethical committee according to the Swiss laws of animal protection and welfare and was licensed under the numbers ZH272/16 and ZH046/20. The project consisted of the currently reported study and concurrent brain imaging studies (30, 31). Informed owner consent was obtained from the owners of the dogs.

### 2.1. Animals

Client-owned dogs of both sexes from the breeds Greater Swiss Mountain Dogs and Border Collies, diagnosed with IE with or without antiseizure drug treatment and their not affected first degree relatives were enrolled into the study. Idiopathic epilepsy was diagnosed based on the International Veterinary Epilepsy Task Force TIER II confidence level (47). They were divided into newly diagnosed dogs without antiseizure drug treatment (IEU) and into dogs already under antiseizure drug treatment (IET). Idiopathic epileptic dogs were only included if they were scanned at least 2 days apart from the last recognized epileptic seizure to limit the possible influence of ictal changes on the spectroscopy (44). As a control group (control) not affected relatives (first degree relative to an epileptic dog but not clinically affected by IE) were included. All healthy relatives were followed up by telephone interviews at the time of writing the manuscript and are still free of seizures (minimum follow up period 2.5 years).

### 2.2. Study design

All dogs were fasted 8–12 h before anesthesia, with water being always accessible. In all dogs, medical history was collected, including information about administration of antiseizure drugs. All patients underwent a clinical and neurological examination performed by a board-certified neurologist. Furthermore, if no recent blood work was available, blood chemistry and hematology were obtained.

^1^H-MRS was performed under general anesthesia. All dogs received 0.2 mg/kg butorphanol (Alvegesic 1% forte ad us. vet., Virbac, Switzerland) either intramuscularly (IM) or IV if a venous catheter (VasoVet, B. Braun Melsungen AG, Germany) had already been placed. The decision of placing a venous catheter into a cephalic vein before or after sedation with butorphanol depended on the behavior of the individual dog. In the Greater Swiss Mountain Dogs 1 mg/kg esomeprazole (Esomep 40 mg, Gruenenthal Pharma AG, Switzerland) was administered IV before induction. All dogs were preoxygenated for 5 min, before induction of general anesthesia with propofol (Propofol 1% MCT Fresenius, Fresenius Kabi AG, Switzerland). The dogs' tracheae were intubated with a cuffed endotracheal tube (Super Safety Clear, Teleflex Medical, Switzerland) of suitable size.

Anesthesia was maintained with sevoflurane (Sevorane, AbbVie AG, Switzerland) to effect, in oxygen and air. The dogs were mechanically ventilated with pressure—controlled ventilation (8–12 cmH_2_O) through a circle system and respiratory rate was adapted to maintain end—tidal CO_2_ between 35 and 38 mmHg. Animals were placed in dorsal recumbency during the ^1^H-MRS, and the following parameters were measured continuously and recorded every 5 min throughout the procedure with a magnetic resonance imaging (MRI) compatible multiparameter monitor (Datex Ohmeda GE N-MRI2-01, Finland): peripheral oxygen saturation, heart rate, non-invasive blood pressure, respiratory rate, end-tidal CO_2_, and end-tidal sevoflurane concentration.

All dogs received an infusion of Ringer's acetate (Ringer Acetate, Fresenius Kabi AG, Switzerland) at rates of 5 ml/kg/h IV. To maintain normotension, which was defined as a mean arterial blood pressure above 60 mmHg five ml/kg of Ringer's acetate were given IV over 10 min as bolus if needed. If up to four fluid boli of Ringer's acetate (5 ml/kg) did not increase the mean arterial blood pressure above 60 mmHg, a dobutamine (Dobutrex 250 mg/50 ml, Teva Pharma AG, Switzerland) constant rate infusion was started at a rate of 0.5 μg/kg/min to increase the mean arterial blood pressure above 60 mmHg.

### 2.3. Spectroscopy data collection and preparation

MRI and MRS of the brain were performed with a 3 T MRI (Philips Ingenia, Philips AG, Switzerland) using a 15 channel receiver transmitter head coil (Stream Head-Spine coil solution, Philips AG, Zurich, Switzerland). Conventional morphological MRI was performed prior to the MRS acquisition. Single voxel MRS acquisition of the right or left thalamus with point resolved spectroscopy (PRESS) was subsequently performed as described in the study of Mauri et al. (31). Briefly summarized T2-W images in all 3 planes were used to place the single voxel in the thalamus, preferably in the right thalamus and carefully avoiding cerebrospinal fluid, as well as peripheral soft and bony tissues adjacent to the thalamus to prevent lipid contamination. Voxel size was 1.8 cm^3^ (10 × 12 × 15 mm). Details of the MRS protocol and analysis are given in the Supplementary Table S1.

**Figure 1:**
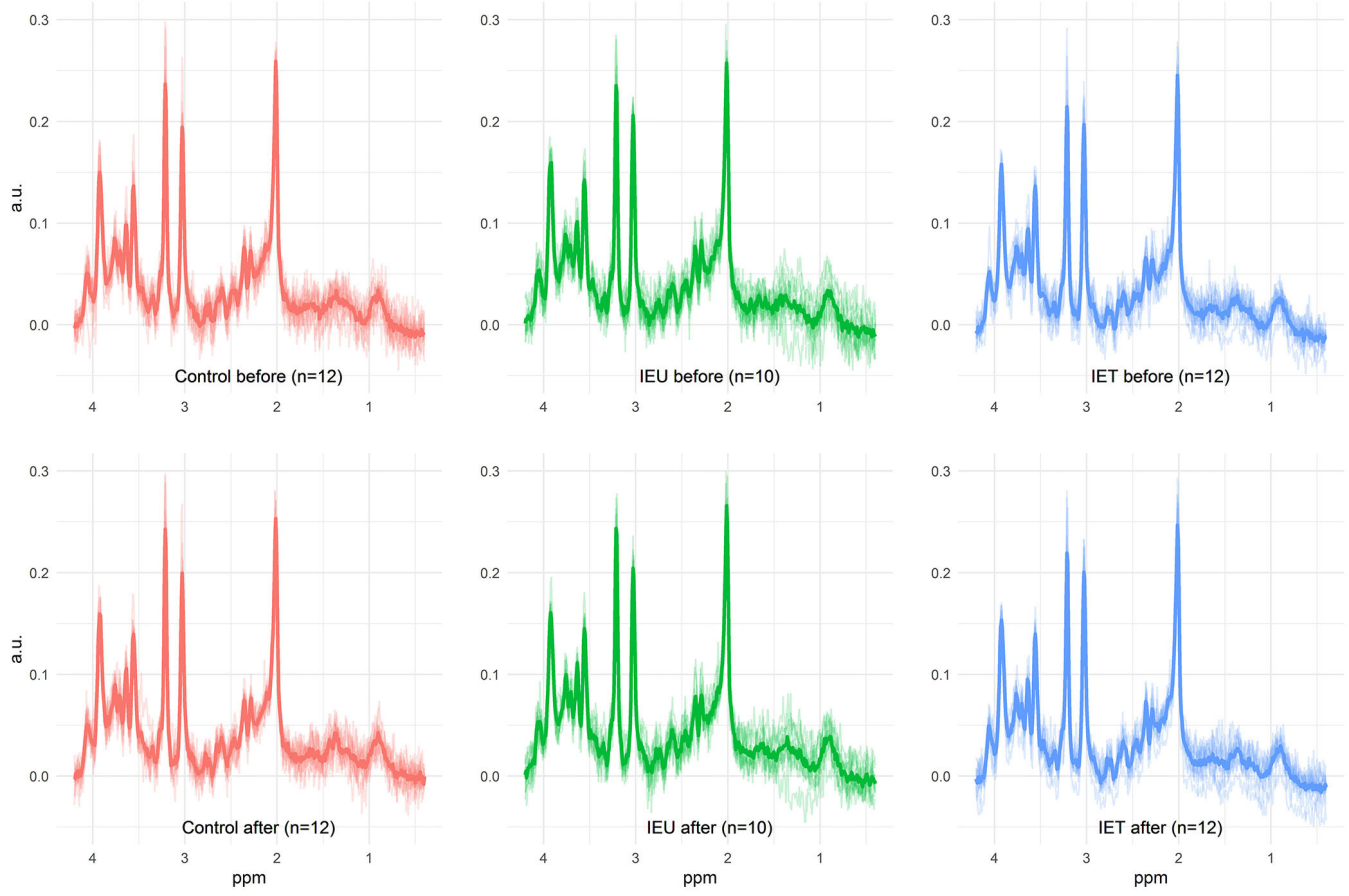
All measured spectra of the three investigated groups prae and post ketamine administration. In the background, the individual spectra of all dogs are plotted as output from LCModel. The thicker line shows the calculated mean spectrum in each group. This figure illustrates the achieved quality of all ^1^H-MRS measurements beyond the quality measures such as SNR and FWHM. In particular, the absence of artifacts can be verified. Metabolite differences described in this study are not visually detectable.

After the first MRS acquisition, 1 mg/kg ketamine was administered IV and 2 min later the MRS scan was repeated. Prior to further analysis the quality of the spectra of the dogs were evaluated. Only spectra of dogs with a morphological normal brain, with correct voxel placement in the thalamus, and with adequate spectral quality (visual inspection for artifacts) were included in our study (Figure 1). The spectral quality in the final data set was assessed based on the signal to noise ratio and the full width at half maximum (FWHM) as given by the software used to analyze the spectra (LCModel, S Provencher, Oakville, ON, Canada) and the full width at half maximum (FWHM) from the unsuppressed water peak.

LCModel was used to estimate metabolite ratios to total creatine and water. Due to a variety of factors contributing to the signal (including not only voxel size, T1 and T2 relaxation times and repetition and echo times, but many more), these measured signals were calibrated against a commonly used reference metabolite (creatine) (48). The ratio of metabolite to total creatine was chosen because tissue segmentation required for correcting the ratio to water was not available in this study (31). Nevertheless, the ratios to water were used to check the results from the ratios to creatine. From all metabolites present in the basis set, only metabolites fitted with a median (over all measurements) relative Cramér Rao lower bound (CRLB) lower than 50 % were included in the following analysis (49).

### 2.4. Statistical analysis and data handling

A mixed ANOVA with the independent factors ketamine administration within subjects and group allocation (control, IEU, IET) between subjects was performed for all measured metabolites, heart rate and mean arterial blood pressure after checking assumptions for normality, outliers and homogeneity of variances and covariances. If significant effects were found, appropriate *post-hoc* tests were run with a Bonferroni correction for multiple comparisons. If the assumptions were not met, we performed a linear mixed effects model. A *p*-value < 0.05 was considered statistically significant.

## 3. Results

Data from 34 dogs fulfilled our quality measures and all quality of spectra were good (Figure 1). These included 12 dogs in the Control group (2 Border Collies and 10 Greater Swiss Mountain Dogs), 10 dogs in the IEU group (2 Border Collies and 8 Greater Swiss Mountain Dogs) and 12 dogs in the IET group (8 Border Collies and 4 Greater Swiss Mountain Dogs). In Table 1 breed, sex, age and weight distribution are presented. The first MRS of 30 of these 34 dogs have been analyzed and published in an independent study (31).

**Table 1:** Sex, median and ranges (minimum and maximum) for age and weight for each group and each breed.

**Breed**	**Control**							**IEU**							**IET**						
	***n*** **(f/m)**	**Age (months)**			**Weight (kg)**			***n*** **(f/m)**	**Age (months)**			**Weight (kg)**			***n*** **(f/m)**	**Age (months)**			**Weight (kg)**		
		**Med**	**Min**	**Max**	**Med**	**Min**	**Max**		**Med**	**Min**	**Max**	**Med**	**Min**	**Max**		**Med**	**Min**	**Max**	**Med**	**Min**	**Max**
BC	2 (1/1)	56	14	98	20.7	19.6	21.8	4 (2/2)	33.5	28	48	18	14	22.7	8 (3/5)	60	45	92	18	15	23
GSMD	10 (6/4)	43.5	21	82	48.5	36	54.8	9 (4/5)	39	11	88	48	32.8	70	4 (1/3)	57.5	9	65	55.5	53	60

Control, healthy siblings; IEU, untreated idiopathic epilepsy; IET, treated idiopathic epilepsy; BC, Border Collie; GSMD, Greater Swiss Mountain Dogs; max, maximum; med, median; min, minimum.

At the timepoint of MRS, dogs in IET group were under one or more antiseizure drugs (mean, minimum and maximum dosages): phenobarbital [3.8 (1.2–4.3) mg/kg BID] in 10 dogs (83%), imepitoin [5.5 (4.3–10) mg/kg BID] in 3 dogs (25%), potassium bromide [16 (14–30) mg/kg BID] in 3 dogs (25%) and / or levetiracetam [15.5 (12–19) mg/kg BID] in 2 dogs (17%), all given orally.

A dobutamine CRI was administered in 10 dogs (7 dogs in the control group, 1 dog in the IEU group and 2 dogs in the IET group) during the ^1^H-MRS scan.

The ^1^H-MRS scan started 77 (55–100) min [median (range)] after propofol administration [4 (2–6) mg/kg]. End-tidal sevoflurane concentration during the scan was 2.3 % (1.8–3.8 %).

All dogs completed the study and recovered uneventfully, except for one dog in the IET group. This dog showed a seizure like phenomenon during the recovery phase and received midazolam 0.5 mg/kg IV as a treatment, which resolved the event.

The spectra of these 34 dogs had a signal to noise ratio of 12.5 (8–20) and a FWHM of 3.96 Hz (2.94–5.88 Hz) according to the LCModel output. The measured FWHM of the water peak was 6.3 Hz (5.4–7.8 Hz). No significant differences in these values were observed between the different groups. The achieved spectral quality is illustrated in Figure 1. Median relative CRLBs were below 50% for the following metabolites: total creatine (sum of creatine and phosphocreatine), the sum of glycerophosphocholine and phosphocholine, the sum of N-acetylaspartate and N-acetylaspartylglutamate, the sum of myo-inositol and glycine, GLX, ascorbate, aspartate, glutathione, and glucose. For GABA, the median relative CRLB was 50.5%. Due to the great interest, GABA was nevertheless analyzed further. The obtained CRLBs are summarized in Supplementary Table S2.

**Figure 2:**
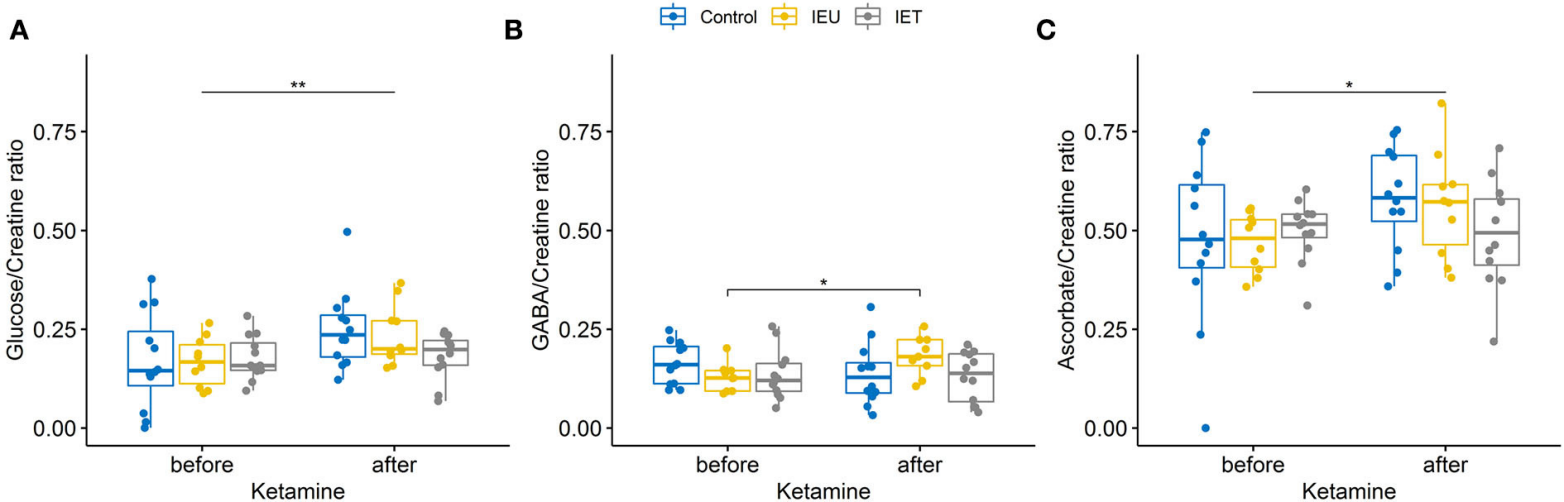
**(A–C)** Boxplot of thalamic metabolite concentrations [**(A)** glucose, **(B)** GABA, **(C)** ascorbate] prae and post ketamine relative to creatine in healthy control dogs (blue), dogs affected by idiopathic epilepsy without antiepileptic drug treatment (IEU in yellow) and treated epileptic dogs (IET in gray). **(A)** Ketamine increased glucose/creatine ratios significantly (*p* < 0.001) but no effect of group allocation was found. A trend for an interaction between group allocation and ketamine administration was observed [p = 0.065, **(A)**]. **(B)** For GABA/creatine ratios no significant effects of ketamine administration (*p* = 0.544) or group allocation (*p* = 0.525) were found, however there was a significant interaction between group allocation and ketamine administration [*p* = 0.038, **(B)**]. *post-hoc* tests revealed a significant increase after ketamine for group IEU (*p* = 0.031). **(C)** For ascorbate a significant effect of ketamine administration could be found using a linear mixed effect model (*p* = 0.033). *p < 0.05, **p < 0.01.

No significant effects of ketamine administration (*p* = 0.356), group allocation (*p* = 0.073) and interaction (*p* = 0.4) for GLX/Creatine were found. However, we found effects on glucose/creatine (Figure 2A), GABA/Creatine (Figure 2B) and ascorbate (Figure 2C).

For all other measured metabolites ratios (sum of glycerophosphocholine and phosphocholine/creatine ratio, the sum of N-acetylaspartate and N-acetylaspartylglutamate/creatine ratio, the sum of myo-inositol and glycine/creatine ratio, aspartate/creatine and glutathione/creatine) no significant effects of ketamine administration, group allocation and interaction were found.

All metabolites' measures (ratio to total creatine) obtained before and after ketamine are summarized in Supplementary Table S3. The results observed in the ratios to creatine were confirmed by the ratios to water signal [glucose/water (*p* < 0.0005) increased significantly with a trend for an interaction between group allocation and ketamine (*p* = 0.061); significant interaction for GABA/water (*p* = 0.035); increase of ascorbate/water using a linear mixed effect model (*p* = 0.036)]. Heart rate, systolic, mean and diastolic blood pressures decreased significantly (all *p* < 0.01) after ketamine administration, while no group or interaction effects could be found.

## 4. Discussion

This study investigated the acute effects of the intravenous administration of 1 mg/kg ketamine on brain metabolites in the thalamus with MRS in dogs suffering from IE with or without antiseizure treatment and in healthy relatives in general anesthesia maintained with sevoflurane. Glucose/creatine ratio increased significantly after ketamine administration in all groups. In contrary to our hypothesis no increase of the excitatory metabolite GLX were found. Interestingly, for the inhibitory metabolite GABA an interaction between ketamine and group allocation was found with IEU showing a significant increase after ketamine administration in comparison to the IET and control group.

### 4.1. Effects on GLX/creatine ratio

Our study did not show any significant effects of ketamine or group allocation on GLX/creatine ratio in the thalamus. Glutamate as the major excitatory neurotransmitter is of special interest in epilepsy and an increased extracellular glutamate in the brain and / or a reduction in GABA concentrations are leading to excitotoxicity, epileptic seizures, and cell death (50).

Former MRS results for glutamate/creatine or GLX/creatine ratio after ketamine administration are limited and controversial, both for people and animals. Reported changes range from an increase to a decrease in glutamate or GLX levels. No changes were found in the GLX/creatine ratio in the thalamus of healthy awake volunteers 2 h after 0.8 mg/kg of ketamine IV administered over 50 min (51). Increased glutamate/creatine ratios were reported in the frontal cortex in healthy rats 30 min after a subanesthetic dose of 30 mg/kg of ketamine subcutaneously (52). Increased levels of glutamine were reported in the anterior cingulate cortex in humans after subanesthetic doses of 0.27 mg/kg ketamine IV followed by a constant rate infusion (CRI) for 2 hours (53). In contrast, decreasing GLX levels were measured in the medial prefrontal cortex of humans suffering from depression with increasing subanesthetic doses of ketamine (0.1–0.5 mg/kg IV) (35).

When looking at the canine literature, higher levels of GLX were reported in the striatum during general anesthesia with 15 mg/kg ketamine IV than during general anesthesia with 8 mg/kg pentobarbital IV (46). However, this study evaluated the absolute difference between these two types of anesthetic drugs in healthy dogs only. Thus, it is not clear if ketamine increased the GLX levels or if pentobarbital decreased them (46). Bektas et al. (manuscript in preparation) evaluated the acute effect of 2 mg/kg ketamine IV on GLX/creatine ratio in the in the hippocampus in healthy Beagle dogs under sevoflurane anesthesia and, in line with our results, did not find any significant changes in GLX/creatine ratios before and after ketamine administration. Comparisons to published studies are difficult due to differences in species and health status, dosage and administration modes of ketamine and variances in timing related to ketamine administration as well as differences in location of the MRS in the brain. There are some possible explanations why we could not detect significant changes in GLX/creatine ratio after ketamine administration.

Firstly, with the current MRS technics applied we were only able to measure GLX/creatine ratio, the sum of glutamine and glutamate. glutamine and glutamate are linked *via* the glutamine–glutamate cycle (54). Changes in the glutamine/glutamate ratios, with an increase in glutamine and a decrease in glutamate have been detected after NMDA—antagonist administration (55). Such changes in opposite directions after ketamine might stay undetected when only the sum of glutamate and glutamine can be measured. To differentiate between glutamate and glutamine a higher magnetic field strength or advanced MRS sequences would be needed.

Secondly, MRS is not able to differentiate between extra—and intracellular metabolites (56). Ketamine is expected to cause an increase of the extracellular glutamate levels (57). Furthermore, the total amount of glutamate is much higher intracellular than extracellular (58). Therefore, a possible extra—or intracellular shift of glutamate after ketamine administration cannot be detected with the current method. As the extracellular levels of glutamate determine the extent of excitation, separation of extra—and intracellular concentrations would be interesting, but currently no non—invasive technique is able to assess such shifts.

Thirdly, we investigated the effect of ketamine administration on the thalamic GLX/creatine ratios only. Since brain regions have different GLX/creatine ratios and different NMDA receptor distribution, it is possible that in other brain areas, changes in GLX/creatine ratios after ketamine administration could have been measured. More studies, with similar doses of ketamine and with voxel placement in different brain areas at higher magnetic field strengths could give us more insights into the effect of ketamine on glutamine and glutamate levels.

In a concurrent study investigating a similar group of dogs the MRS of the thalamus before ketamine showed decreased GLX/creatine ratios in the treated IE dogs compared to Controls and untreated IE dogs (31). The current study shows that, although changes in GLX/creatine ratio may be present in IET dogs, their reaction to a low dose of ketamine in general anesthesia seems comparable to their healthy and their untreated IE relatives.

### 4.2. Effects on glucose/creatine ratio

The detected increase in glucose/creatine ratio in MRS could have two possible explanations. Firstly, ketamine could have increased the supply of glucose to the brain by increasing the blood glucose level, by decreasing the regional metabolic rate of glucose or by increasing the brain perfusion (59). Secondly, ketamine could have decreased brain glucose consumption (60). The decreased consumption is possibly caused by a direct NMDA blockade (54, 61) or indirectly by an increased anesthetic depth (62). Decreases in heart rate and blood pressure after ketamine in our study indicate deeper plane of anesthesia. Ketamine and deeper plane of anesthesia lead to vasodilation (63) with a possible concurrent increase in blood supply to the brain.

In people, a bolus of 0.5 mg/kg of ketamine IV showed reduction or increases of the regional metabolic rate of glucose in different brain regions (64). In contrast, a target-controlled ketamine infusion (0.3 μg/ml) led to an overall increase of regional metabolic rate of glucose in the whole brain, with the highest increase in the thalamus (65). However, another study revealed no change after a target-controlled s-ketamine infusion (0.75 μg/ml) of regional metabolic rate of glucose at all (66). In rats, high intraperitoneal dosages of ketamine (170 mg/kg) decreased regional metabolic rate of glucose in the thalamus in one study (67), whereas in another study 10–30 mg/kg of ketamine IV lead to different results according to the brain area, with no effect measured in the thalamus (68).

In human IE patients, glucose consumption is reduced inter-ictally and elevated ictally compared to baseline (69, 70). We did not detect different glucose/creatine response after ketamine administration between IE dogs and healthy control dogs. However, a trend for an interaction between group allocation and ketamine administration was found (Figure 2A) and the effect of ketamine on glucose/creatine ratio was most prominent in the IEU dogs. Interestingly, the dog with the reported seizure like phenomenon during recovery had the lowest increase in glucose/creatine. Whether this low increase of glucose/creatine ratio had been caused by an already ongoing silent epileptic seizure activity during the MRS or if it is a coincidental finding cannot be further elucidated.

Not only the regional metabolic rate of glucose, but also the blood glucose concentration can be changed by ketamine administration and can therefore be a reason for a change in brain glucose/creatine ratio. In rabbits, a low dose of 0.17 mg/kg of ketamine IV produced hyperglycemia and higher doses of 1–2 mg/kg IV produced hypoglycemia (71). In dogs, no elevation in blood glucose after a ketamine bolus of 1 or 2 mg/kg IV could be detected in one study (72). However, another study documented an elevation in blood glucose using 10 mg/kg of ketamine IV (73), so, a dose dependent elevation in blood glucose can be assumed. Unfortunately, blood glucose levels were not measured in the current study, which presents a limitation to discuss further what caused the elevated glucose/creatine ratios in the thalamus. Further studies are needed to explain the exact mechanism between ketamine and glucose/creatine ratio in the brain. Studying different dosages and different brain regions might give further insights into this finding.

### 4.3. Effects on GABA/creatine ratio

Measuring GABA using ^1^H-MRS is challenging due to the low concentration of GABA in the brain and the spectral overlap with other metabolites. Thus, GABA levels should be interpreted with caution, unless measured at higher magnetic field strength (≥7 T MRS) or special sequences (74). Even though we found a significant increase of GABA/creatine ratio in group IEU, the median relative CRLB were high, so the current results should be interpreted with care. In future studies the measurement of GABA in dogs could be optimized, although the voxel size is limited due to the small size of our patients' brain. Using spectral editing sequences such as MEGA-PRESS with longer scan times and correction for B_0_ instability would enable more reliable GABA levels (75).

Ketamine administration blocks the postsynaptic NMDA receptor, therefore more glutamate can bind to pre-synaptic inhibitory glutamate receptors (76). When these receptors are activated by glutamate binding, a retrograde messenger is released, which can result in an increase in GABA (20, 77) and could further explain the increase in GABA/creatine ratio after ketamine administration. An increase in the GABA/creatine ratio could indicate a neuroprotective effect of ketamine in the epileptic dogs, by shifting the balance toward more inhibitory metabolites in the brain (12, 50).

Furthermore, lower GABA concentrations have been found in the urine in untreated IE dogs compared to treated IE dogs and healthy controls (32). Interestingly, ketamine reverses the GABA deficit in human major depression patients (35) which may also apply to untreated epileptic dogs. In epileptic patients, GABA turnover can be reduced, with not yet fully understood reasons (69). A reduced glycolysis and therefore a decrease in glutamate turnover in inter-ictal IE patients has been reported as a possible reason. As glutamate can be transformed into GABA *via* the glutamic acid decarboxylase, a decrease in glutamate turnover can lead to a decrease in GABA (69). It is possible that dogs treated with GABAergic anti-epileptic drugs, have GABA concentrations in the brain, that are more similar to concentrations in healthy individuals, which could be shown in studies in human medicine (41, 78, 79) and which is supported by the recent study from Mauri et al. (31). Furthermore, increases in GABA levels were reported in the medial prefrontal cortex after 0.5 mg/kg over 40 min of ketamine IV in humans suffering from obsessive—compulsive disorder (33). Possibly ketamine reverses the GABA deficit reported in untreated canine IE patients (51, 80).

### 4.4. Effects on ascorbate/creatine

Not only an increase in glucose/creatine ratio, but also a significant increase in ascorbate/creatine ratio was detected after ketamine administration. Ascorbate is an important antioxidant in the brain that can be reliably detected using MRS at 3 T field strength (81, 82) and resonates at 3.73 ppm which is close to the GLX compounds resonation (81). To preserve antioxidant effects, ascorbate underlies strict homeostasis in the brain (82). Increases in extracellular glutamate concentrations have been associated with increases in ascorbate concentrations in the brain under experimental conditions (83, 84). While the effect of ketamine on brain ascorbate levels has not been investigated, premedication with ascorbate before ketamine anesthesia has shown to enhance the effect of ketamine anesthesia in rabbits (85). In animal epileptic seizure models, administration of a high dose of ascorbate has shown anti–seizure and neuroprotective effects, but it is unknown whether ascorbate regulation is impacted by natural occurring epilepsy (86). No differences between groups have been found in the current study.

It is difficult to transfer the current findings into clinical implications as no studies comparing metabolite estimates and clinical effects in dogs are available. In this study we could prove that the effect of a drug on brain metabolites can be measured non-invasively using ^1^H-MRS in clinical patients. This opens the door for future studies exploring the effect of antiseizure medication in epileptic dogs. Possible indication might be the investigation of drug resistance in epilepsy and to elucidate how ketamine interrupts refractory status epilepticus in dogs.

### 4.5. Limitations

In the current study all animals were under general anesthesia and metabolite concentrations can be altered due to anesthetic choice of drugs (45, 87). It is therefore debatable if the findings after administration of ketamine under general anesthesia can be translated to the administration of ketamine in awake patients. To minimize the interaction of anesthetic drugs during the current study, we chose short acting drugs for premedication (butorphanol) and induction (propofol). For maintenance of anesthesia, we chose sevoflurane, as a recent study found that sevoflurane had only minor effects without clinical relevance on GLX concentrations (45).

We decided to use 1 mg/kg ketamine IV as this dosage is often used as co-induction agent and also for analgesic purposes (88). As we used a lower dose than the commonly used doses for status epilepticus treatment in dogs and because our dogs were scanned inter-ictally we cannot draw direct conclusions about the effects of higher dosages of ketamine in ictal dogs (10).

Antiseizure drug treatment can result in chronic changes in brain metabolite concentrations (89). The effects might differ between different drugs, different epileptic syndromes, and different brain areas (79, 90). The dogs included in our study were not on a standardized treatment and a variety of drugs in different dosages were used, precluding a definitive conclusion about the effect on antiseizure drug treatment on the MRS results.

As a further limitation electroencephalography (EEG) of the IE dogs was not available. Clinically no epileptic seizures were reported at least 2 days before the MRS scan for all IE dogs, but the absence of epileptic seizures was not confirmed by EEG and underlying epileptic seizure activity could have been missed. Additionally, we cannot prove the absence of any ictal activity during MRS scans, because of the lack of EEG during the MRS scan.

The volume of interest was chosen in the thalamus, due to the region's involvement in IE in humans (91) and dogs (92) and the presence of NMDA-receptors in the thalamus. As IE is not only involving the thalamus and ketamine is acting also in other parts of the brain (hippocampus, cortex), the effects of ketamine in other parts of the brain could be different and should be investigated in future studies by examining different brain regions.

The sample size was relatively small with groups not balanced regarding breeds, age and sex. However, the sex distribution with more males than female in IEU and IET dogs reflects the natural distribution of the disease in dogs, with a higher risk of epilepsy in male dogs (93, 94). Further studies assessing differences among breeds are needed to exclude a possible bias.

Some dogs were treated with dobutamine to maintain non-invasive mean arterial blood pressure above 60 mmHg. However, no change of rate before and after the ketamine administration was performed, so a possible influence of dobutamine would not interfere with our study results.

Increase of GABAergic transmission can be also due to acute or chronic stress (95). Dogs are commonly stressed in veterinary environment (96), but we did not investigate stress levels of the individual dogs to account for stress as a possible cofounder.

## 5. Conclusion

^1^H-MRS was able to detect changes in metabolite/creatine ratios after intravenous administration of 1 mg/kg ketamine IV in dogs. We found no evidence of excitatory effects induced in the thalamus. A slight difference in the response to ketamine was detected between IEU dogs and healthy control and IET dogs. Although it is beyond the scope of this study to investigate the antiseizure potential of ketamine in dogs, results of this research suggest that the effect of ketamine on the brain metabolites in dogs could be dependent on the concentrations of brain metabolites before administration.

## Data availability statement

The original contributions presented in the study are included in the article/Supplementary material, further inquiries can be directed to the corresponding author.

## Ethics statement

The animal study was reviewed and approved by the Cantonal Authorities according to Swiss Law Under Animal License Nos. ZH272/16 and ZH046/20. Written informed consent was obtained from the owners for the participation of their animals in this study.

## Author contributions

RB and KB conceived the project. NZ made technical contributions to the ^1^H MRS protocol and accomplished MRS data sampling and analyzing, supported by NM and MW. RB, AK, HR, and MW participated in interpretation of the study results. MW drafted the paper and was supported by KB, RB, and AK. The manuscript was revised by KB, AK, NZ, HR, NM, and RB. All authors read and approved the final manuscript.
