# Supplementary material for: Ketamine administration in idiopathic epileptic and healthy control dogs: Can we detect differences in brain metabolite response with spectroscopy?
Source: Front Vet Sci. 2023 Jan 6;9:1093267. doi: 10.3389/fvets.2022.1093267 (PMC9853535; doi:10.3389/fvets.2022.1093267)
Supplement: Supplementary file 1 [file Data_Sheet_1.PDF]

Supplementary table 1: Details of the single voxel ^{1}H-MRS protocol

1. Hardware	
a. Field strength [T]	3 T
b. Manufacturer	Philips
c. Model (software version if available)	Ingenia (R 5.2)
d. RF coils: nuclei (transmit/receive), number of channels, type, body part	15 channel receive-transmit head coil (Stream Head-Spine coil solution)
e. Additional hardware	N/A
2. Acquisition	
a. Pulse sequence	PRESS
b. Volume of Interest (VOI) locations	Thalamus
c. Nominal VOI size [cm^3, mm^3]	10 x 12 x 15 mm^3 (APxRLxFH)
d. Repetition Time (TR), Echo Time (TE) [ms, s]	TR 2000 ms, shortest TE : 31 ms
e. Total number of Excitations or acquisitions per spectrum	240 acquisitions per spectrum
f. Additional sequence parameters (spectral width in Hz, number of spectral points, frequency offsets)	Spectral width 2000 Hz, 1024 points. Frequency offset of the RF Pulses was set to the frequency of Creatine.
g. Water Suppression Method	Excitation
h. Shimming Method, reference peak, and thresholds for "acceptance of shim" chosen	Second-order automatic pencil-beam shim
i. Triggering or motion correction method<br><br>(respiratory, peripheral, cardiac triggering, incl. device used and delays)	N/A
3. Data analysis methods and outputs	

a. Analysis software	LCModel (Version 6.3)
b. Processing steps deviating from quoted reference or product	Processing of .sdat /. spar provided by Philips with a)Residual water filter ; b) 1Hz Gaussian Filter c) FWHM of water peak calculation
c. Output measure<br><br>(e.g. absolute concentration, institutional units, ratio)	Ratio to total creatine and to water. The ratio to water is obtained with the LCModel defaults for WCONC and ATTH20, see below. With that it is a rough estimate of moles of metabolite per volume of brain tissue (mol/L).
d. Quantification references and assumptions, fitting model assumptions	An unsuppressed water peak was acquired with the same TR and TE as the metabolites and with the offset frequency set to water.<br><br>The unsuppressed water peak was used as reference with WCONC= 43300 and ATTH20=0.7 set within in LCModel. 20 Metabolites included in the simulated basis set (alanine, aspartate, glucose, creatine, phosphocreatine, glutamine, glutamate, glycerophosphocholine, phosphocholine,lactate, mI, NAA,N-acetyl-aspartyl-glutamate, scyllo-inositol, glutathione, taurine, glycine, phosphoethanolamine , ascorbate, and γ-aminobutyric acid.<br><br>Simulated contribution of macro molecules and lipid signals provided within LCModel.
4. Data Quality	
a. Reported variables<br><br>(SNR, Linewidth (with reference peaks))	SNR (Output from LCModel), FWHM of the water peak
b. Data exclusion criteria	Voxel placement outside of the thalamus, visual inspection for artifacts
c. Quality measures of postprocessing Model fitting (e.g. CRLB, goodness of fit, SD of residual)	%CRLBs of selected Metabolites
d. Sample Spectrum	Figure 2
