# Supplementary material for: Ketamine administration in idiopathic epileptic and healthy control dogs: Can we detect differences in brain metabolite response with spectroscopy?
Source: Front Vet Sci. 2023 Jan 6;9:1093267. doi: 10.3389/fvets.2022.1093267 (PMC9853535; doi:10.3389/fvets.2022.1093267)
Supplement: Supplementary file 2 [file Data_Sheet_2.pdf]

Supplementary table S2: ratios to Creatine of all measured metabolites

Metabolite	Control				IEU				IET			
	prae		post		prae		post		prae		post	
	mean	SD	mean	SD	mean	SD	mean	SD	mean	SD	mean	SD
GPC.PCh	0.36	0.05	0.37	0.03	0.36	0.05	0.37	0.03	0.33	0.06	0.32	0.05
NAA. NAAG	1.21	0.17	1.17	0.1	1.19	0.12	1.21	0.07	1.11	0.13	1.12	0.13
mI.Gly	1.18	0.11	1.2	0.09	1.15	0.1	1.17	0.07	1.16	0.11	1.14	0.07
NAA	1.02	0.13	0.98	0.08	0.99	0.17	1.03	0.11	0.93	0.07	0.93	0.1
Glu.Gln	1.78	0.22	1.71	0.3	1.81	0.14	1.69	0.22	1.59	0.19	1.64	0.15
mI	1.01	0.12	1	0.16	0.93	0.12	0.94	0.08	0.93	0.16	0.89	0.1
Glu	1.33	0.19	1.31	0.16	1.29	0.11	1.24	0.18	1.2	0.21	1.22	0.14
Asc	0.48	0.21	0.58	0.13	0.47	0.07	0.56	0.13	0.5	0.08	0.49	0.14
Asp	0.46	0.13	0.46	0.14	0.45	0.11	0.49	0.07	0.46	0.1	0.46	0.1
NAAG	0.19	0.07	0.2	0.06	0.2	0.1	0.18	0.08	0.18	0.07	0.19	0.07
GSH	0.18	0.1	0.14	0.09	0.17	0.05	0.16	0.04	0.14	0.05	0.15	0.06
Gln	0.45	0.08	0.4	0.22	0.52	0.14	0.45	0.13	0.39	0.13	0.41	0.1
Glc	0.17	0.12	0.25	0.1	0.17	0.06	0.23	0.08	0.18	0.06	0.18	0.06
PE	0.32	0.25	0.19	0.12	0.17	0.09	0.19	0.14	0.24	0.13	0.24	0.14
Scyllo	0.03	0.02	0.02	0.01	0.02	0.02	0.02	0.02	0.02	0.02	0.03	0.02

GABA	0.16	0.05	0.14	0.08	0.15	0.07	0.19	0.05	0.14	0.06	0.13	0.06
Gly	0.17	0.16	0.21	0.09	0.21	0.11	0.23	0.12	0.23	0.16	0.25	0.12
Lac	0.09	0.1	0.1	0.08	0.04	0.04	0.06	0.06	0.06	0.05	0.07	0.08
Tau	0.05	0.07	0.05	0.13	0.04	0.05	0.03	0.04	0.03	0.03	0.06	0.08
Ala	0.03	0.04	0.05	0.05	0.06	0.06	0.07	0.06	0.05	0.06	0.06	0.07

Ala	alanine
Asc	ascorbate
Asp	aspartate
Crea	creatine
GABA	gamma aminobutyric acid
Glc	glucose
Glu.Gln	the sum of glutamine and glutamate
Gly	glycine
GPC.PCh	the sum of glycerophosphocholine and phosphocholine
GSH	Glutathione
IET	idiopathic epilepstic dogs treated with anti-epileptic drug treatment
IEU	idiopathic epileptic dogs without anti-epileptic drug treatment
Lac	lactate
mI	myo-inositol
mI.Gly	the sum of myo-inositol and glycine
NAA	N-acetylaspartate
NAAG	N-acetylaspartylglutamate
NAA.NAAG	the sum of N-acetylaspartate and N-acetylaspartylglutamate

PCh	phosphorylcholine
PE	phosphorylethanolamine
Scyllo	scylloinositol
SD	standard deviation
Tau	taurine
