# Supplementary material for: Ketamine administration in idiopathic epileptic and healthy control dogs: Can we detect differences in brain metabolite response with spectroscopy?
Source: Front Vet Sci. 2023 Jan 6;9:1093267. doi: 10.3389/fvets.2022.1093267 (PMC9853535; doi:10.3389/fvets.2022.1093267)
Supplement: Supplementary file 3 [file Data_Sheet_3.pdf]

Supplementary table S3: details Cramer-Rao-Lower-Bounds

%CRLB metabolite	Control						IEU						IET					
	prae			post			prae			post			prae			post		
	med	min	max	med	min	max	med	min	max	med	min	max	med	min	max	med	min	max
GPC.PCh	4	3	5	4	3	4	4	3	6	4	3	5	3	3	5	3	3	4
NAA. NAAG	4.5	3	5	4	3	5	4	3	6	4	3	5	4	3	5	4	3	5
mI.Gly	5	3	6	4.5	3	5	4.5	3	7	5	3	6	3.5	3	6	3.5	3	5
NAA	5	4	6	5.5	4	7	5	4	9	5	4	7	5	4	7	4	4	8
Glu.Gln	7.5	6	9	8	6	12	7.5	5	9	8	5	12	7	5	10	6.5	5	9
mI	8.5	5	16	9	6	13	10.5	6	15	10.5	6	13	7	5	16	7.5	5	14
Glu	9	6	12	8	7	12	9	6	12	9.5	6	14	8	6	14	7.5	6	11
Gln	26	19	35	31.5	15	75	21.5	17	31	25.5	19	40	26	15	41	22	16	39
Asp	24	15	68	26.5	15	34	25.5	16	45	24.5	16	37	20.5	16	35	20	15	34

NAAG	24.5	16	61	27	15	51	28	13	48	24	18	150	23	16	66	21	15	53
GSH	25.5	14	109	31.5	18	999	30.5	20	36	29.5	19	94	25	21	70	28	14	60
GABA	48	31	84	60	27	275	54	26	77	39	27	84	53.5	25	122	46.5	28	176
Asc	19.5	12	999	16.5	10	32	22	13	31	18	11	32	16.5	12	26	15	10	33
Scyllo	41.5	25	999	66	40	999	89.5	24	999	67	27	999	59.5	12	999	44	14	999
Glc	33.5	17	999	24.5	15	45	32.5	14	88	27.5	14	46	32	17	53	30.5	16	62
PE	40	13	999	49.5	25	999	63	34	999	61	26	999	45.5	16	999	37.5	18	999
Gly	84	27	999	41	16	263	46	18	119	31.5	15	693	30	17	999	27.5	19	86
Lac	90.5	32	999	69	36	999	241	58	999	243	40	999	138	39	999	84	35	999
Tau	697	40	999	999	18	999	553.5	66	999	519	68	999	291.5	83	999	273.5	30	999
Ala	711.5	67	999	172.5	62	999	104	44	999	111.5	54	999	137.5	39	999	110.5	31	999

Ala	alanine
Asc	ascorbate
Asp	aspartate

Crea	creatine
CRLB	Cramer-Rao-Lower-Bound
GABA	gamma aminobutyric acid
Glc	glucose
Glu.Gln	the sum of glutamine and glutamate
Gly	glycine
GPC.PCh	the sum of glycerophosphocholine and phosphocholine
GSH	Glutathione
IET	idiopathic epileptic dogs treated with anti-epileptic drug treatment
IEU	idiopathic epileptic dogs without anti-epileptic drug treatment
Lac	lactate
max	maximum
med	median
mI	myo-inositol
mI.Gly	the sum of myo-inositol and glycine
min	minimum
NAA	N-acetylaspartate
NAAG	N-acetylaspartylglutamate
NAA.NAAG	the sum of N-acetylaspartate and N-acetylaspartylglutamate

PCh	phosphorylcholine
PE	phosphorylethanolamine
Scyllo	scylloinositol
SD	standard deviation
Tau	taurine
